# Knowledge and Awareness of Drug-Induced Hypersensitivity Reactions Among Nurses and Pharmacists: A Cross-Sectional Study

**DOI:** 10.7759/cureus.92369

**Published:** 2025-09-15

**Authors:** Waleed M Altowayan, Abdulmajeed Alqasoumi, Abdulrhman I Mohana, Abdulrahman Alsowinea, Mohammed S Alkathlan, Talal S Nheet, Turki A Alharbi, Alwleed Alaidah, Fahad M Alharbi, Ahmed M ALRashidi, Khaled A Alkhalawi

**Affiliations:** 1 Department of Pharmacy Practice, College of Pharmacy, Qassim University, Buraydah, SAU; 2 Communicable Disease, Public Health Authority, Riyadh, SAU; 3 Department of Medicine, College of Medicine, Qassim University, Buraydah, SAU; 4 Infectious Diseases, King Fahad Specialist Hospital, Buraydah, SAU; 5 Pharmacy, Al-Dawaa Pharmacies, Riyadh, SAU; 6 Pharmacy, Nahdi Pharmacies, Riyadh, SAU

**Keywords:** anaphylaxis, cross-sectional study, drug allergy, healthcare professionals, hypersensitivity reaction, knowledge assessment, nurses, pharmacists, saudi arabia

## Abstract

Background

Drug-induced hypersensitivity reactions (DHRs), including drug allergies and anaphylaxis, present significant risks in clinical practice. Prompt recognition and appropriate management are vital for patient safety. However, knowledge gaps among healthcare professionals can lead to misdiagnosis and improper treatment. This study aimed to assess the knowledge of healthcare workers regarding the mechanisms, diagnosis, and management of drug allergies.

Methods

A cross-sectional study was conducted between 2023 and 2024 across eight hospitals in the Al-Qassim region, Saudi Arabia. A validated, self-administered questionnaire comprising demographic items and 14 knowledge questions was distributed to pharmacists and nurses (n=169). Descriptive statistics summarized the participant characteristics and response patterns. Independent t-tests, one-way ANOVA, and Chi-square tests were used to assess differences and associations, with a significance level set at p<0.05.

Results

The participants were predominantly female respondents (n=119, 70.4%) and nurses (n=124, 73.4%), with most holding a bachelor’s degree (n=138, 81.7%). The mean knowledge score was 6.36±2.23 (range: 2-14), and the mean precision score was 45.39±15.96 (range: 14.29-100). No significant differences in mean scores were found by gender, job title, education level, or hospital level. Correct response rates ranged from 15 (8.9%) for identifying the indication for a drug provocation test to 148 (87.6%) for identifying penicillin as the recommended drug for intradermal testing. Significant associations were found between specific knowledge items and demographic variables, including job title (p=0.020), gender (p=0.001), education level (p=0.040), and hospital level (p<0.001).

Conclusion

The overall knowledge and precision scores among healthcare professionals were suboptimal, with notable gaps in diagnostic and procedural aspects of drug allergy management. Targeted educational interventions are recommended to address these gaps, particularly focusing on diagnostic procedures and appropriate clinical responses.

## Introduction

Drug allergies are a significant concern in clinical practice due to their impact on patient safety, treatment outcomes, and healthcare costs [1]. As a subset of adverse drug reactions (ADRs), drug allergies are immune-mediated responses that can lead to mild cutaneous symptoms or progress to life-threatening conditions such as anaphylaxis [2,3]. These hypersensitivity reactions are commonly classified into immediate and delayed types, with Type I hypersensitivity, mediated by IgE and mast cells, being the most well-known cause of anaphylaxis and other rapid-onset allergic reactions [4,5]. Healthcare workers (HCWs), including nurses and pharmacists, play a critical role in the early identification, documentation, and management of drug-induced hypersensitivity reactions. Accurate knowledge of drug allergy mechanisms, clinical manifestations, and appropriate diagnostic and therapeutic interventions is essential to reduce the risk of morbidity and mortality [6]. However, evidence suggests that many HCWs possess inadequate understanding of these reactions, which may lead to misdiagnosis, delayed treatment, or unnecessary drug avoidance [7,8].

Although prior studies have assessed general knowledge and preparedness for managing ADRs in various regions of Saudi Arabia [9], there is limited data specifically addressing drug allergy awareness among healthcare professionals in the Qassim region. A study at King Abdulaziz Medical City in Riyadh involving 373 HCWs (64% nurses, 25% pharmacists) revealed low overall knowledge regarding drug hypersensitivity reactions; less than one-third reported consistently inquiring about patient allergy history, and only 42% felt that more advanced training was necessary [10]. Another survey focused on penicillin allergy awareness among healthcare providers in Riyadh found that only 45% were satisfied with their level of knowledge, while more than 70% acknowledged that penicillin allergy negatively impacted quality of life [11]. These findings underscore both the knowledge deficit and the willingness to learn among HCWs in Saudi settings.

Across Saudi Arabia, gaps in pharmacovigilance understanding and ADR reporting are also well documented. Hospital-based studies have shown that many practitioners, particularly nurses, lack familiarity with the definition of ADRs and the principles of pharmacovigilance, which impedes both reporting and proactive management of allergic reactions [12]. Educational interventions have shown efficacy: a trial of structured ADR education among hospital pharmacists demonstrated a significant improvement in knowledge scores after a three-month intervention [13]. The study primarily assessed the knowledge of healthcare workers regarding drug-induced hypersensitivity reactions, with secondary aims of evaluating response accuracy and examining associations between knowledge and demographic factors. Identifying knowledge gaps will inform the development of targeted educational interventions and clinical guidelines to enhance the safety and quality of care in patients with suspected or confirmed drug allergies.

## Materials and methods

Study design and setting

This cross-sectional study was conducted between 2023 and 2024 in the Al-Qassim region of Saudi Arabia. Data were collected from eight hospitals, including King Fahad Specialist Hospital, Buraydah Central Hospital, Alras General Hospital, King Saud Hospital, Alamal and Psychiatric Hospital, Uyun Al Jawa General Hospital, Alsyah Hospital, and Dr. Suliman Alhabib Hospital. The study was approved by the Qassim Cluster Research Committee prior to data collection.

Study population

The study targeted HCWs, specifically pharmacists and nurses, who were actively practicing in their respective roles at the selected hospitals. There were no restrictions regarding years of experience or specialty, as the objective was to assess a broad understanding of drug allergy knowledge across professional levels. Participants were eligible if they were working in one of the included hospitals and provided informed consent to participate in the study.

Sampling and sample size

A total of 169 healthcare professionals were recruited using a non-probability convenience sampling method. The sample included individuals from both secondary and tertiary healthcare facilities to ensure diverse representation of the healthcare workforce in the region.

Data collection tool

A structured, self-administered questionnaire was adapted from the knowledge domain of a previously validated instrument developed by Wang et al. [14], used under the terms of the Creative Commons Attribution-NonCommercial license (CC BY-NC 4.0). The original tool consisted of 25 items across three domains (knowledge, attitudes, and practices). For this study, only the 14 knowledge-based multiple-choice items were retained to align with the study objectives. These items covered drug allergy classification, clinical manifestations, diagnostic approaches, and management strategies. The questionnaire also included four demographic items tailored to the Saudi healthcare setting: gender, education level, job title (nurse or pharmacist), and hospital level (secondary, tertiary, or community). Each knowledge item was presented as a multiple-choice question with a single correct answer, scored as 1 for correct and 0 for incorrect. The total knowledge score (range 0-14) reflected the sum of correct responses, and a knowledge precision score (percentage) was calculated as the proportion of correct responses relative to the total possible ones. The questionnaire was distributed in person by trained members of the research team, who obtained written informed consent prior to completion and were available to clarify questions without influencing responses.

Data collection procedures

The electronic version of the questionnaire was developed using Google Forms (Google, California, US). The form was shared with eligible participants in person by members of the research team, either through direct device access or by providing a secure link during hospital visits. Before completing the form, participants were informed about the study objectives and procedures, and written informed consent was obtained. The research team remained available to clarify any questions but did not influence participant responses. All responses were submitted electronically and stored securely within the Google Forms platform, then exported to a password-protected database for analysis. Anonymity and confidentiality were maintained throughout the data collection process

Data analysis

The collected data were entered and analyzed using the IBM SPSS Statistics for Windows, Version 26 (Released 2019; IBM Corp., Armonk, New York, United States). Descriptive statistics, including frequencies, percentages, means, and standard deviations, were used to summarize participant demographics and knowledge responses. A knowledge precision score was calculated to assess overall performance in the knowledge section. Inferential statistical analysis was conducted using independent t-tests to examine differences between the two groups, such as gender and job title. One-way analysis of variance (ANOVA) or Chi-square tests were used where comparisons involved more than two categories, such as education level and hospital type. A p-value of less than 0.05 was considered statistically significant.

Ethical considerations

This study adhered to ethical principles in accordance with the Declaration of Helsinki. Ethical approval was obtained from the Regional Research Ethics Committee, Qassim Province (approval no: 607/44/10971). All participants were informed of the study’s purpose, and written informed consent was obtained from each participant before data collection. Participation was voluntary, and anonymity and confidentiality were maintained throughout the research process.

## Results

Participant characteristics

A total of 169 healthcare professionals participated in this study, comprising 50 male respondents (29.6%) and 119 female respondents (70.4%). The majority held a bachelor’s degree (n=138, 81.7%), followed by diploma holders (n=19, 11.2%), and those with a master’s degree or board certification (n=12, 7.1%). Nurses accounted for 124 participants (73.4%), while pharmacists represented 45 participants (26.6%). More than half of the respondents were from tertiary hospitals (n=86, 50.9%), followed by secondary hospitals (n=75, 44.4%) and community centres (n=8, 4.7%) (Table 1).

**Table 1:** Demographic characteristics of the participants (n=169) Values are presented as frequencies (n) and percentages (%).

Variable	Category	n	%
Gender	Male	50	29.6
	Female	119	70.4
Education	Diploma	19	11.2
	Bachelor	138	81.7
	Master/Board & above	12	7.1
Job title	Pharmacist	45	26.6
	Nurse	124	73.4
Hospital level	Tertiary	86	50.9
	Secondary	75	44.4
	Community health center	8	4.7

Knowledge and precision scores

The mean knowledge score was 6.36±2.23 (range: 2-14), while the mean precision score was 45.39±15.96 (range: 14.29-100). Independent t-tests revealed no statistically significant differences in knowledge scores between the male and female participants (p=0.837) or between pharmacists and nurses (p=0.586). One-way ANOVA showed no significant association between knowledge or precision scores and education (p=0.277) or hospital (p=0.065) levels (Table 2).

**Table 2:** Knowledge and precision scores by demographic characteristics Values are presented as mean ± standard deviation (SD); p-values are from independent t-tests (for comparisons between two groups: gender, job title) or one-way analysis of variance (ANOVA) (for comparisons between the three groups: education level, hospital level). A p-value of <0.05 was considered statistically significant.

Variable	Group	Mean knowledge score ± SD	Mean precision score ± SD	t/F value	p-value
Gender	Male	6.30 ± 2.71	45.00 ± 19.37	t = -0.207	0.837
	Female	6.38 ± 2.01	45.56 ± 14.38		
Job title	Pharmacist	6.51 ± 2.70	46.51 ± 19.30	t = 0.546	0.586
	Nurse	6.30 ± 2.05	44.99 ± 14.63		
Education	Diploma	6.95 ± 2.53	48.84 ± 15.72	F = 1.293	0.277
	Bachelor	6.33 ± 2.21	45.05 ± 15.99		
	Master/Board & above	6.08 ± 2.19	41.96 ± 16.79		
Hospital level	Tertiary	6.81 ± 2.20	48.98 ± 15.77	F = 2.787	0.065
	Secondary	6.13 ± 2.08	43.49 ± 15.81		
	Community	5.50 ± 3.12	39.29 ± 19.06		

The distribution of participant responses for each knowledge item is presented in Table 3.

**Table 3:** Frequencies and percentages of participant responses for each item of the questionnaire (n=169) All response options are presented as frequencies (n) and percentages (%).

Item No.	Question & response options	n	%
1	Drug-induced anaphylaxis belongs to which type of hypersensitivity?		
	Type I (Correct answer)	120	71
	Type II	22	13
	Type III	10	5.9
	Type IV	17	10.1
2	All of the following factors are related to drug allergy, EXCEPT:		
	Drug dosage (Correct answer)	38	22.5
	Drug exposure	16	9.5
	Administration route	34	20.1
	Heredity	81	47.9
3	Which is the effector cell in drug-induced anaphylaxis?		
	Mast cell (Correct answer)	98	58
	Lymphocyte	35	20.7
	Eosinophils	31	18.3
	Monocytes	5	3
4	When will immediate drug hypersensitivity reactions occur after drug administration?		
	<6 h (Correct answer)	128	75.7
	6-8 h	28	16.6
	8-12 h	9	5.3
	12-24 h	4	2.4
5	Which antibody mediates immediate drug hypersensitivity reactions?		
	IgE (Correct answer)	123	72.8
	IgG	28	16.6
	IgM	14	8.3
	IgA	4	2.4
6	What is the most common clinical manifestation of drug allergy?		
	Anaphylaxis	124	73.4
	Cutaneous symptoms (Correct answer)	40	23.7
	Serum sickness	2	1.2
	Hepatic and renal injury	3	1.8
7	What is regarded as the gold standard for diagnosing drug allergy?		
	Provocation tests (Correct answer)	21	12.4
	Clinical history	30	17.8
	Skin tests	101	59.8
	Drug-specific IgE	17	10.1
8	What is the indication for a drug provocation test?		
	The suspected drug is imperative or cannot be replaced for the concurrent illness (Correct answer)	15	8.9
	Suspected drug allergy	89	52.7
	Suspected drug allergy associated with systemic disease	38	22.5
	Suspected drug allergy with serious cutaneous symptoms	27	16
9	Which test is recommended as the first screening step when immediate drug hypersensitivity is suspected?		
	Skin prick test (Correct answer)	76	45
	Intradermal test	35	20.7
	Skin patch test	18	10.7
	Provocation test	40	23.7
10	Appropriate time for allergy confirmation by skin test when drug allergy is suspected?		
	Anytime	93	55
	Right after symptoms disappear	47	27.8
	At least 1 month after symptoms disappear (Correct answer)	16	9.5
	Never	13	7.7
11	In which situation is penicillin skin test NOT appropriate?		
	Recent Tylenol (Correct answer)	70	41.4
	Recent dexamethasone	53	31.4
	Recent leukotriene modifier	12	7.1
	Recent aminophylline	34	20.1
12	Which drug is recommended for intradermal test?		
	Penicillin (Correct answer)	148	87.6
	Aztreonam	6	3.6
	Ofloxacin	6	3.6
	Azithromycin	9	5.3
13	Key management strategy for drug allergy?		
	Drug therapy	53	31.4
	Specific immunotherapy	18	10.7
	Symptomatic therapy	26	15.4
	Avoiding sensitization drugs (Correct answer)	72	42.6
14	First-choice medication in anaphylactic shock?		
	Dopamine	21	12.4
	Antihistamine	29	17.2
	Glucocorticoid	10	5.9
	Epinephrine (Correct answer)	109	64.5

Chi-square tests identified several statistically significant associations (Table 4).

**Table 4:** Significant associations between knowledge items and demographics (Chi-square tests) Only statistically significant associations are presented; χ²: Chi-square statistic; p-values are based on Pearson Chi-square test; a p-value <0.05 was considered statistically significant.

Knowledge Item (correct answer)	Demographic Variable	χ²	p-value
Drug-induced anaphylaxis type (Type I)	Job title	5.38	0.02
All of the following factors related to drug allergy, EXCEPT drug dosage	Gender	12.50	<0.001
	Job title	13.71	<0.001
	Hospital level	11.85	0.003
Which is the effector cell in drug-induced anaphylaxis (Mast cell)	Education	6.45	0.04
	Hospital level	9.28	0.01
When immediate drug hypersensitivity reactions occur (<6 h)	Hospital level	8.86	0.012
Which antibody mediates immediate drug hypersensitivity reactions (IgE)	Job title	5.06	0.025
	Hospital level	13.73	0.001
Indication for drug provocation test (suspected drug allergy)	Gender	10.87	0.001
	Job title	13.51	<0.001
Which test recommended as first screening step (skin prick test)	Gender	6.48	0.011
Appropriate time for allergy confirmation by skin test (At least 1 month after symptoms disappear)	Education	8.73	0.013
Inappropriate situation for penicillin skin test (recent dexamethasone, Tylenol, etc.)	Gender	6.96	0.008
	Education	10.00	0.007
	Hospital level	7.27	0.026
First-choice medication in anaphylactic shock (Epinephrine)	Hospital level	21.83	<0.001

Job title was significantly associated with correctly identifying drug-induced anaphylaxis as a type I hypersensitivity reaction (p=0.020) and with knowledge of the antibody mediating immediate drug hypersensitivity (p=0.025). Gender, job title, and hospital level were all significantly associated with the question on factors related to drug allergy (all p<0.005). Education level and hospital type were significantly associated with knowledge of the effector cell in drug-induced anaphylaxis (p=0.040 and p=0.010, respectively). Hospital level was also significantly associated with knowledge on the timing of immediate hypersensitivity reactions (p=0.012), the antibody mediating immediate hypersensitivity (p=0.001), and the correct first-choice medication in anaphylactic shock (p<0.001). Additionally, gender and job title were significantly related to knowledge of drug provocation test indications (p=0.001 and p<0.001, respectively), while education level was associated with the correct timing for allergy confirmation by skin testing (p=0.013).

## Discussion

This study evaluated HCWs' knowledge of drug-induced hypersensitivity reactions, particularly drug allergies and anaphylaxis. The findings revealed knowledge gaps in several critical areas, including the pathophysiology of hypersensitivity reactions, diagnostic approaches, and management strategies. These results are consistent with those of prior international studies that have highlighted insufficient awareness among healthcare providers regarding drug allergy mechanisms and appropriate interventions [15,16]. A significant proportion of participants correctly identified drug-induced anaphylaxis as a Type I hypersensitivity reaction mediated by IgE and mast cells. However, misconceptions regarding other types of hypersensitivity were evident. Similar gaps in knowledge have been reported in previous studies, in which healthcare professionals often confused the mechanisms underlying different hypersensitivity reactions [10,17]. The incorrect attribution of anaphylaxis to Type II, III, or IV hypersensitivity suggests the need for targeted educational interventions, as delayed-type reactions (Type IV) are often mistaken for immediate hypersensitivity [18].

The study found that only a small percentage of participants (22.5%) correctly identified that drug dosage was not a key factor in drug allergy development. This misconception aligns with the findings of previous research, in which healthcare providers frequently overestimated the role of dosage in hypersensitivity reactions [19]. While drug exposure and administration route influence allergic responses, drug allergies are primarily immune-mediated rather than dose dependent [20]. Regarding clinical manifestations, most participants (73.4%) incorrectly identified anaphylaxis as the most common symptom of drug allergy, whereas the correct answer, cutaneous symptoms, was chosen by only 23.7%. This aligns with the findings of a previous study, which reported that non-allergist or specialized practitioners often fail to recognize early cutaneous manifestations of drug allergy, leading to misdiagnosis or delayed treatment [21].

A knowledge gap was observed in the diagnostic approaches. Provocation tests are the gold standard for diagnosing drug allergies [22], yet only 12.4% of participants identified this correctly. Instead, 59.8% incorrectly believed skin tests were the gold standard. Previous studies have reported similar trends, with healthcare providers underutilizing skin tests and over-relying on clinical history [23]. Moreover, the proper indication for a drug provocation test, that the suspected drug is imperative or cannot be replaced, was correctly recognized by only 8.9% of respondents. This finding is in line with that of Popiolek et al., who emphasized that many physicians avoid provocation testing due to concerns about safety, despite its critical role in confirming or ruling out drug hypersensitivity [24].

Another major concern was the timing of the allergy testing. Less than 10% of the participants correctly identified that allergy confirmation should be performed at least one month after symptom resolution. This is particularly important because premature testing can lead to false-negative results due to immune system suppression [25]. Thus, follow-up protocols or policies were not explored. In terms of management, (42.6%) of the participants correctly identified drug avoidance as the key strategy for managing drug allergies. However, a significant proportion incorrectly believed that symptomatic therapy or specific immunotherapy was the primary management approach. Although symptomatic treatments, such as antihistamines and corticosteroids, can alleviate allergic symptoms, they do not prevent recurrent reactions [26]. Regarding the treatment of anaphylaxis, only 64.5% of the participants correctly identified epinephrine as the first-choice medication. This finding is concerning because epinephrine is the only effective treatment for anaphylactic shock, and delays in administration increase the risk of fatality [27]. The analysis of the knowledge precision scores revealed no significant differences based on gender, educational level, or hospital type. However, Chi-square analysis showed significant associations between specific knowledge items and demographic variables, including gender, job title, education level, and hospital level, indicating that certain subgroups performed better on specific questions. This aligns with previous findings that demographic factors may influence topic-specific knowledge rather than overall performance [28,29]. Given that nurses often serve as first responders in allergic emergencies, enhancing their knowledge through targeted educational programs remains crucial.

This study had several limitations. First, the use of a cross-sectional design limits the ability to infer causality between knowledge and demographic variables. Second, the sample was obtained using a non-probability convenience sampling method, which may have introduced selection bias and limited the generalizability of the findings to all HCWs in the Qassim region or other regions in Saudi Arabia. Third, the study relied on self-administered questionnaires, which may be subject to response bias, as participants may have provided socially desirable answers. Additionally, the questionnaire did not assess actual clinical practices or behaviors, which may differ from the theoretical knowledge. Despite these limitations, this study provides valuable insights into existing knowledge gaps and highlights the need for targeted educational interventions.

## Conclusions

This study revealed that healthcare professionals in the Al-Qassim region, including both pharmacists and nurses, demonstrated suboptimal knowledge and precision scores regarding drug allergy classification, clinical manifestations, diagnosis, and management. Significant gaps were identified in diagnostic practices, recognition of appropriate testing procedures, and understanding of critical management steps for severe reactions. While some associations between knowledge and demographic characteristics were observed, these findings highlight the need for targeted, profession-specific educational interventions. Strengthening training in drug allergy recognition and management may enhance patient safety and improve clinical outcomes.

## Appendices

Questionnaire on drug allergy knowledge and awareness

**Table 5:** Questionnaire used in the study Adapted from [14]; licensed under CC By NC 4.0 (http://creativecommons.org/licenses/by-nc/4.0/).

Section 1: Demographics		
Sr. No.	Question	Options
1	Gender	A. Male B. Female
2	Education level	A. Diploma B. Bachelor’s C. Master’s/Board certified
3	Job title	A. Nurse B. Pharmacist
4	Type of healthcare facility	A. Secondary B. Tertiary C. Community Health Center
Section 2: Drug Allergy Questionnaire		
Q#	Question	Options
A1	Drug-induced anaphylaxis belongs to:	A. Type I hypersensitivity B. Type II hypersensitivity C. Type III hypersensitivity D. Type IV hypersensitivity
A2	All of the following are related to drug allergy, EXCEPT:	A. Drug dosage B. Drug exposure C. Administration route D. Heredity
A3	Which is the effector cell in drug-induced anaphylaxis?	A. Mast cells B. Lymphocyte C. Eosinophils D. Monocytes
A4	When do immediate drug hypersensitivity reactions occur after drug administration?	A. <6 hr B. 6–8 hr C. 8–12 hr D. 12–24 hr
A5	Which antibody mediates immediate drug hypersensitivity reactions?	A. IgE B. IgG C. IgM D. IgA
A6	What is the most common clinical manifestation of drug allergy?	A. Anaphylaxis B. Cutaneous symptoms C. Serum sickness D. Hepatic and renal injury
A7	What is regarded as the gold standard to diagnose drug allergy?	A. Clinical history B. Skin tests C. Drug-specific IgE D. Provocation tests
A8	What is the indication for drug provocation test?	A. Suspected drug allergy B. Suspected allergy with systemic disease C. Drug is imperative or irreplaceable D. Allergy with serious cutaneous symptoms
A9	Which test is recommended as the first screening step for immediate hypersensitivity reactions?	A. Skin prick test B. Intradermal test C. Skin patch test D. Provocation test
A10	When should allergy confirmation by skin test be performed after symptoms disappear?	A. Anytime B. Right after symptoms disappear C. At least one month after symptoms disappear D. Never
A11	In which case is penicillin skin testing inappropriate?	A. Treated with paracetamol/cold meds recently B. Treated with IV dexamethasone yesterday C. On leukotriene modifier in last 3 days D. Treated with aminophylline recently
A12	Which of the following drugs requires intradermal testing before administration?	A. Penicillin B. Aztreonam C. Ofloxacin D. Azithromycin
A13	What is the pivotal management strategy for drug allergy?	A. Drug therapy B. Specific immunotherapy C. Symptomatic therapy D. Avoiding sensitization drugs
A14	Which medication is the first choice in anaphylactic shock?	A. Dopamine B. Antihistamine C. Glucocorticoid D. Epinephrine
